# Molecular network of important genes for systemic sclerosis-related progressive lung fibrosis

**DOI:** 10.1186/s13104-015-1510-4

**Published:** 2015-10-07

**Authors:** Yan Jiao, Hong Chen, Tianshu Gu, Lishi Wang, Arnold Postlethwaite, Weikuan Gu

**Affiliations:** Departments of Orthopedic Surgery and BME, Campbell Clinic, University of Tennessee Health Science Center, 956 Court Avenue Rm A302, Memphis, TN 38163-0001 USA; Mudanjiang Medical College, Mudanjiang, 157001 Heilongjiang People’s Republic of China; The First Hospital of Qiqihaer City, 30 Gongyuan Road, Longsha District, Qiqihaer, 161005 Heilongjiang People’s Republic of China; Hebei Medical University, Shijiazhuang, 050011 Hebei People’s Republic of China; Department of Medicine, University of Tennessee Health Science Center, Memphis, TN 38163-0001 USA; Department of Veterans Affairs Medical Center, Memphis, TN 38104 USA

**Keywords:** Gene network, Human, Mouse, Lung fibrosis, Systemic sclerosis

## Abstract

**Background:**

Considerable progress has been made in illuminating the pathological events for systemic sclerosis (SSc)-related progressive lung fibrosis. The molecular events that lead to SSc-related progressive lung fibrosis need to be defined. Some important genes have been identified from a recent study in humans. We aim to construct and compare the similarities and differences of molecular pathways between SSc-related progressive lung fibrosis and normal lungs of humans and mice.

**Methods:**

We used the analytical approach of association of key genes in SSc-related progressive lung fibrosis. We first identified the probes for genes of SSc-related progressive lung fibrosis and analyzed the pathways in human lung using data generated by microarray. We then analyzed the gene pathways in mouse lung for similar sets of probes. Gene expression data from livers were used to compare with that in lung in both humans and mice.

**Results:**

Our analysis indicated that, in humans, the expression levels of genes for macrophage activation are more strongly associated with each other than that in mice. In both humans and mice, the associations of these genes are much greater in the lung than that in the liver. The association in gene expression between humans and mice are similar for IFN-regulated genes and profibrotic/Tgfβ-regulated genes.

**Conclusion:**

Our analysis reveals the differences and similarities of the network of key genes between humans and mice during the molecular processes that eventually lead to fibrosis in the lung.

**Electronic supplementary material:**

The online version of this article (doi:10.1186/s13104-015-1510-4) contains supplementary material, which is available to authorized users.

## Background

Pulmonary fibrosis is now one of the leading causes of death from systemic sclerosis (SSc). Studies on molecular mechanisms of SSc-related progressive lung fibrosis have been conducted in animal models as well as in human populations. A recent publication reported a set of genes that are differentially expressed between SSc-related progressive lung fibrosis and a control healthy population in humans [1]. This set included genes associated with macrophage activation and chemokine, IFN, and profibrotic/TGFβ-regulated gene expression. These genes are potentially important for the diagnosis and for understanding of the molecular mechanisms related to progressive SSc lung fibrosis. Whole genome gene expression profiles from mouse recombinant inbred (RI) strains and human populations have been widely used for the construction of gene network to illustrate the potential molecular pathways of genes of interests [2, 3]. Therefore, construction of gene network of these differentially expressed genes may enhance our understanding of their molecular pathways and regulatory connections.

This study aims to explore the potential pathways that connect these genes in human lung and to investigate whether similar pathways exist in mice. The identification of these differentially expressed genes for progressive SSc lung fibrosis is a very important step for eventual understanding of the molecular events in this manifestation of SSc. Moreover, understanding molecular mechanisms of changes of gene expression in SSc lung fibrosis will greatly enhance our ability to select targets for drug design. One important step in understanding the mechanism of these changes is to determine how these genes are regulated and related to each other under normal genomic conditions. Understanding the network of these genes in a normal genomic background will provide information on how these genes are regulated in pathways dysregulated in SSc lung fibrosis. This knowledge will enhance our understanding on how the gene pathways are altered and how the disease is trigged, and therefore, what and how the targets should be selected for drug design. In this study, we will focus on the construction of pathways of these genes under the normal genomic background using data from normal lungs of humans and mice. Other diseases may sometimes affect gene expression of similar sets by different mechanisms or genes at alternate, but similar, steps in the pathways. We will also compare networks between humans and mice so as to provide information on similarities and differences to guide studies of murine models of lung fibrosis.

## Methods

### Human gene expression data sets

GeneNetwork expression data from lung were based on results of 1230 samples [4], which contain gene expression levels of the whole-genome from human lung tissues processed with Affymetrix HuRSTA array. The gene expression data are available through GSE23546 at the GeneNetwork (http://www.genenetwork.org/webqtl/main.py).

The expression level of genes from human liver is from the Human Liver Cohort (HLC) study, which aimed to characterize the genetic architecture of gene expression in human liver using genotyping, gene expression profiling, and enzyme activity measurements of cytochrome P450. The HLC was assembled from a total of 780 screened liver samples [5, 6]. Data on gene expression of 427 samples are used in this study.

The Whole-genome gene expression profiles of non-tumorous human lung tissues in GeneNetwork is contributed by the Genotype-Tissue Expression (GTEx) Project. The data set currently includes RNA sequence data from 119 individuals from three data sets of whole-genome gene expression profiles of non-tumorous human lung tissues: Laval set (GSE23352), UBC set (GSE23529), and GRNG set (GSE23545). RNA-seq was performed using the Illumina TruSeq library construction protocol [7, 8].

### Mouse gene expression data sets from recombinant inbred strains

Gene expression data from the mouse lung in recombinant inbred (RI) strains and standard inbred strains was obtained with Affy Mouse Genome 430 2.0 (GPL1261) [7] (http://www.genenetwork.org/webqtl/main.py?FormID=sharinginfo&GN_AccessionId=160). The data set includes the whole gene expression profiles from 61 mouse strains, including 47 RI strains from BXD (derived from C57BL/6J and DBA/2J), two parents, two F1s, and ten standard inbred strains.

Gene expression data from mouse liver was from the data set of GSE16780 UCLA Hybrid MDP Liver Affy HT M430A, which was performed by Dr. Lusis’ group at UCLA (http://www.genenetwork.org/webqtl/main.py?FormID=sharinginfo&GN_AccessionId=373). The data set includes the whole genome expression profiles of 99 mouse strains, including 29 RI strains from BXD, 12 RI strain form AXB (A/J and C57BL/6J), 12 RI strains from BXA (C57BL/6J and A/J), and 11 RI strains form BXH (C57BL/6J and C3H/HeJ). Remaining strains are standard inbred strains.

### Genes for evaluation

We include three groups of genes from a previous study [3] that were shown to be differentially expressed in SSc fibrotic lung. The first group are the genes for macrophage activation [including CD163 (cluster of differentiation 163), AIF-1 (allograft inflammatory factor 1), CD86 (cluster of differentiation 86), and MS4A4A (membrane-spanning 4-domains, subfamily A, member 4A)] and chemokines potentially contributing to leukocyte infiltration [including CCL18 (chemokine [C–C motif] ligand 18), CCL13 (chemokine [C–C motif] ligand 13), CXCL5 (chemokine [C–X–C motif] ligand 5), and CCR1 (chemokine [C–C motif] receptor 1)].

The second group is IFN-regulated genes which includes IFNAR2 (IFN-α β and Ω, receptor 2), OAS1 (2-prime,5-prime-oligoadenylate synthetase 1), IL-18 [interleukin 18 (IFNβ-inducing factor)], and TLR7 (Toll-like receptor 7), IFI44 (interferon-induced protein 44), OAS2 (2-prime,5-prime oligoadenylate synthetase 2), and MX1 [myxovirus resistance 1, IFN-inducible protein p78 (mouse)].

The third group is the profibrotic/Tgfβ-regulated genes. They are collagen genes, COL5A2 (collagen type V, α2), COL3A1 (collagen type III, α1), and COL1A1 (collagen type I, α1) COL14A1 (collagen type XIV, α1) as well as SPP1 (secreted phosphoprotein 1/osteopontin) and COMP (cartilage oligomeric matrix protein).

Genes involved in IGF signaling, especially proteins with high affinity for IGF, such as IGFBP-3 and IGFBP-5, as well as low-affinity IGF binding proteins, such as IGFBP-7, CTGF and cyr61, were also aberrantly expressed in SSc diseased lungs and fibroblasts.

### Analysis methods

Information related to expression level and gene association and network were all analyzed using the GeneNetwork [9]. Based on the list of genes, the searching key words for 23 genes are “CD163, AIF-1, CD86, MS4A4A, CCL18, CCL13, CXCL5, CCR1, IFN, IFNAR2, OAS1, IL-18, TLR7, IFI44, OAS2, MX1, Tgfβ, COL5A2, COL3A1, COL1A1, COL14A1, SPP1, COMP”. Probes are collected and selected based on the list of the genes. In case of multiple probes, we first used all the probes in the construction of the gene network. If they are all highly positively correlated. Then one probe (usually the one with the highest expression level) is used for the final construction of the gene network. Graphic connections and associations within the gene network are accomplished with the Metrix and Network Graphs at GeneNetwork. Initial edge lengths were computed by applying an r-to-Z transform to the correlation coefficients and then inverting the results. The graph drawing algorithm found a configuration that minimizes the total stretching of the edges. Curves show Pearson correlation coefficients >0.35 or <−0.35. The graph’s canvas is 40.0 by 40.0 cm, and the node labels are drawn with a 10.0 point font, and the edge labels are drawn with a 10.0 point font.

A graph based on literature correlation was constructed by selecting the commend of literature in the graphic section. Based on GeneNetwork, the literature correlation is defined as a measure of the similarity of words used to describe genes. Sets of words that are associated with genes are compared using latent semantic indexing methods. Sets of words associated with genes are extracted from MEDLINE/PubMed abstracts (http://www.genenetwork.org).

## Results

### Gene relative expression levels of multiple probes of 23 genes

We first examined whether one probe could represent a gene in case multiple probes are obtained. When we searched the probes of 23 genes from the human lung databases, we initially found 82 probes. These 82 probes (Additional file 1: Table S1) represent the 23 genes and their closely relevant genes; however, they are also the results of multiple probes of single genes amongst these genes [10]. For the purpose of simplicity and clarity, we used the probe of the highest expression level in case there were multiple probes for a gene. We examined whether one probe represent the others of the same gene in the 23 genes, we initially conducted the gene network analysis of the genes with multiple probes. The result indicated that different probes of the same genes are highly significantly associated (Additional file 2: Figure S1). Therefore we eliminated the multiple probes of genes, leaving each gene with one probe of the highest the expression level for the analysis.

### Gene network of systemic sclerosis-related genes in lung and liver in humans

**Fig. 1:**
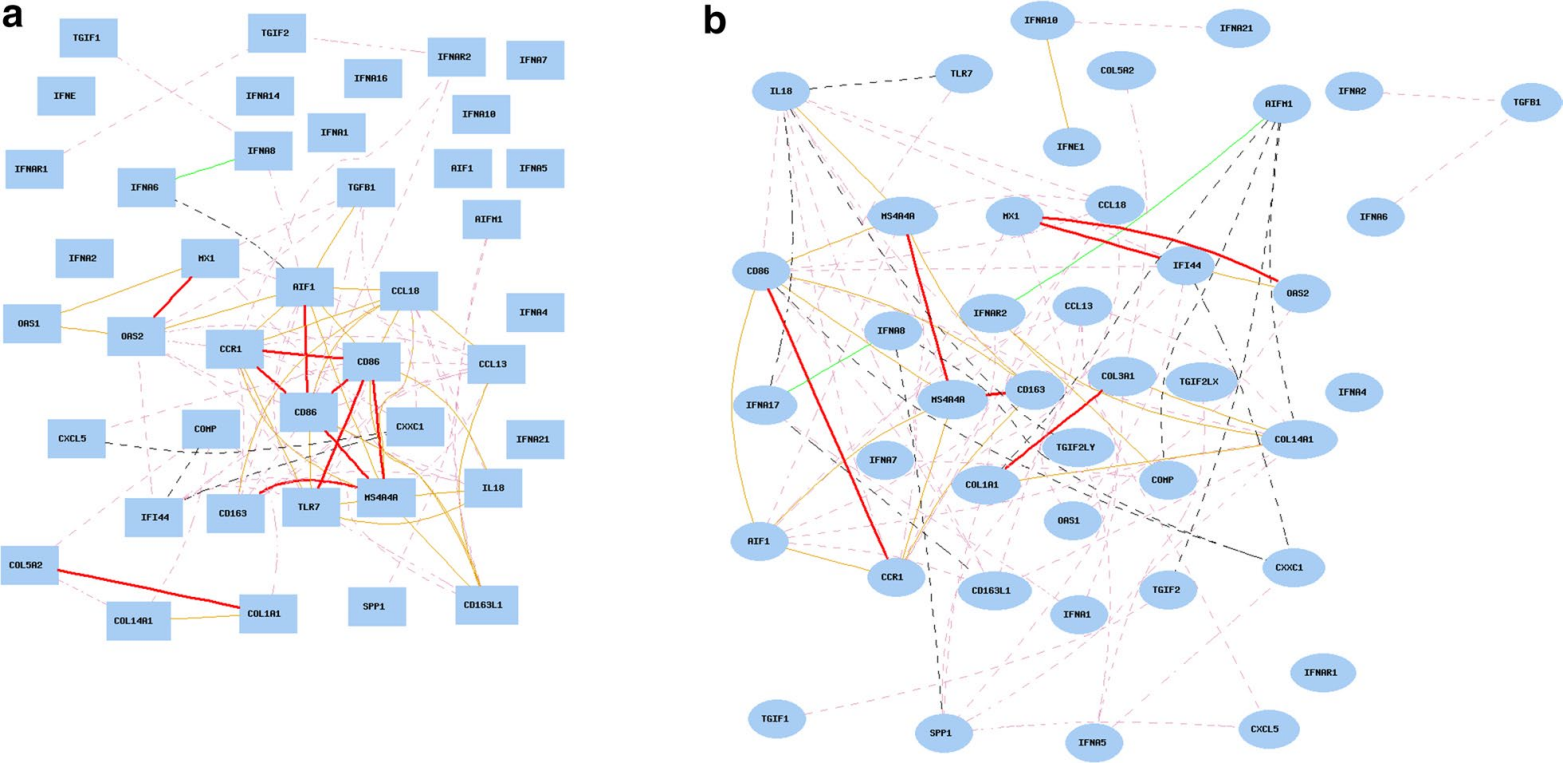
Gene network of SSc-related genes in humans. Curves show Pearson correlation coefficients >0.35 or <−0.35. The graph’s canvas is 40.0 by 40.0 cm, and the node labels are drawn with a 10.0 point font, and the edge labels are drawn with a 10.0 point font. *Colors* of *lines* represent different R values. *Red* 0.7–1, *pink* 0.5–0.7, *grey pink* 0–0.5, *black* −0.5 to 0, *green* −0.7 to −0.5, *blue* −1 to −0.7. **a** Gene network of SSc-related genes in lung of humans. The 41 nodes in the graph below show the selected traits. All nodes are displayed. The 89 edges between the nodes, filtered from the 820 total edges are drawn as curves. **b** Gene network of SSc-related genes in liver of humans The 42 nodes in the graph below show the selected traits. All nodes are displayed. The 85 edges between the nodes, filtered from the 861 total edges are drawn as curves

We then analyzed the network of genes in human lung. After eliminating the multiple probes, we obtained 42 probes, each representing one of the 23 genes and their closely relevant genes (Additional file 3: Table S2). Gene network construction indicated that the expression levels of majority of these genes are positively associated (Fig. 1a). In particular, genes for macrophage activation (CD163, AIF1, CD86, MS4A4A, CCL18, CCL13, and CCR1) are positively associated—most of them with R values >0.7. CXCL5, however, showed a weak association with CD86.

The other group of strongly associated genes is Profibrotic/Tgfβ-regulated genes including COL5A2, COL1A1, and COL14A1 which are strongly and positively associated. COMP is weak but positively associates with each of the three genes above. However, SPP1 is only weakly positively associated with COL3A1.

IFN-regulated genes do not seem to be closely associated as a whole group. MX1, OAS1, and OAS2 are associated together, and their expression seems connected to macrophage activation through AIF1. IL-18 and TLR7 positively associated with each other and to macrophage activation through CD86 and MS4A4A. There is no direct regulation of this group of genes by either IFN or IFNAR2.

The gene expression network in liver was analyzed in the same way as in the lung. Gene network construction indicated that the expression levels of a majority of these genes are positively associated (Fig. 1b), however, not as strongly as that in lung. Genes for macrophage activation (CD163, AIF1, CD86, MS4A4A, and CCR1) positively associated together; but CCL13, CCL18, and CXCL5 exhibited much weaker or no association with these five genes.

Among IFN-regulated genes, OAS1, IFI44, OAS2, and MX1 are associated as a group. IL-18 is positively connected to the macrophage activation group through MS4A4A.

Profibrotic/Tgfβ-regulated gene expressions had no obvious associations amongst each other.

These results suggest that, in the lung, the major driving force for fibrosis is the genes for macrophage activation and Profibrotic/Tgfβ-regulated genes. However, the associations of these genes in liver are not as strong as in the lung—possibly indicating these molecular events are limited to SSc-related progressive lung fibrosis in case of the mutation of COL3A1 in human.

### Gene network of systemic sclerosis-related genes in lung and liver in mouse

**Fig. 2:**
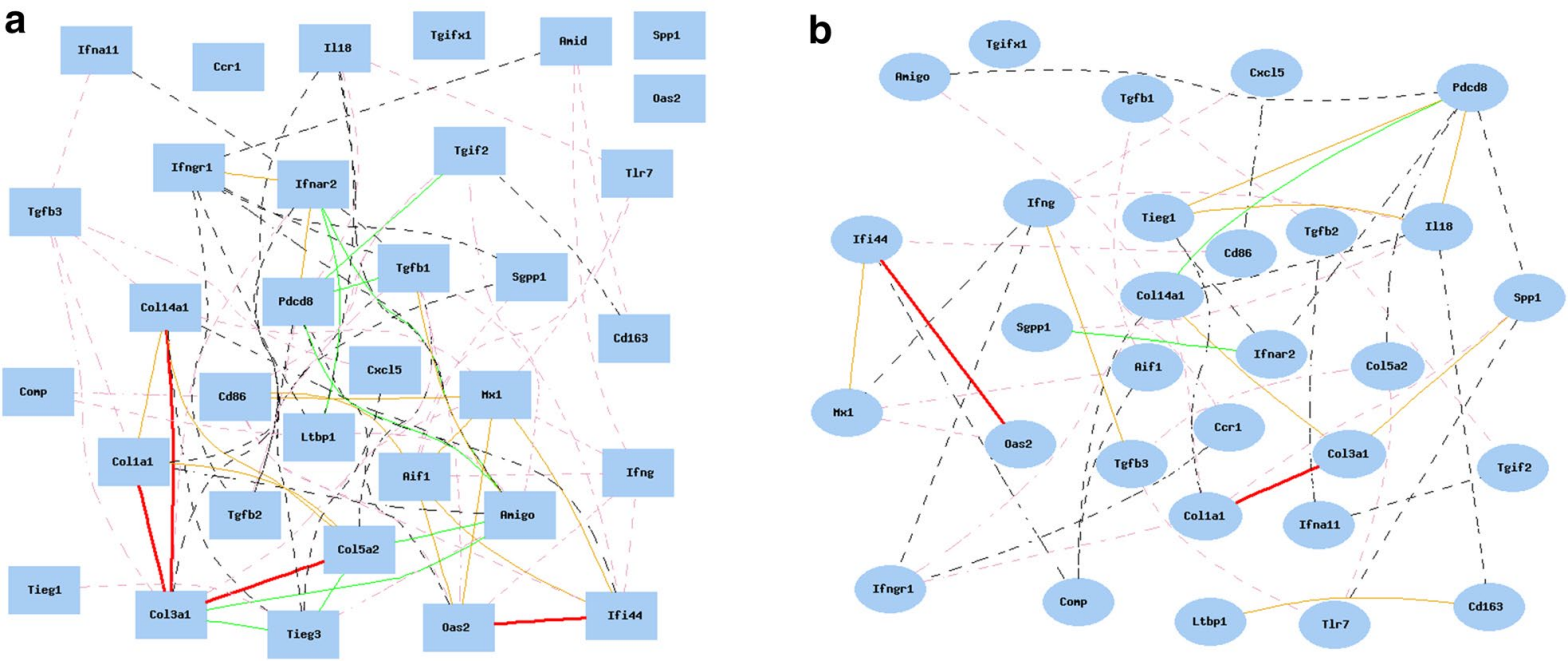
Gene network of SSc-related genes in BXD RI strains. Curves show Pearson correlation coefficients >0.35 or <−0.35. The graph’s canvas is 40.0 by 40.0 cm, and the node labels are drawn with a 10.0 point font, and the edge labels are drawn with a 10.0 point font. *Colors* of *lines* represent different R values. *Red* 0.7–1, *pink* 0.5–0.7, *grey pink* 0–0.5, *black* −0.5 to 0, *green* −0.7 to −0.5, *blue* −1 to −0.7. **a** Genenetwork of SSc-related genes in lung of BXD RI strains. The 33 nodes in the graph below show the selected traits. All nodes are displayed. The 84 edges between the nodes, filtered from the 528 total edges are drawn as curves. **b** Genenetwork of SSc-related genes in liver of BXD RI strains. The 30 nodes in the graph below show the selected traits. All nodes are displayed. The 48 edges between the nodes, filtered from the 435 total edges are drawn as curves

From the gene expression data of mouse lung, we identified 66 probes. After eliminating the duplicated probes, probes for 33 genes were used for the analysis of the gene network (Fig. 2). The strongly associated genes in mouse lung are collagen synthesis genes in the group of profibrotic/*Tgfβ*-regulated genes*. Col14a1*, *Col3a1*, *Col1a1*, and *Col5a2* are strongly positively associated with each other. However, *Tgfβ*, *Comp*, and *Spp*1 did not show association with these collagen synthesis genes.

Among IFN-regulated genes, *Mx1*, *Oas2*, and *Ifi44* are associated together as one group, and their expression is also positively associated with that of *Aif1* and *Cd86* (both are considered as genes in macrophage activation). However, *Aif1* and *Cd86* are not associated with other genes in macrophage activation. *IL*-*18* and *Tnip1*, *Pdcd8*, *Ifnar2,* and *Ifnar1* are positively associated. For unknown reasons, *Tlr7* did not show any strong association with any of these genes.

Except for *Aif1* and *Cd86*, there is no association among genes for macrophage activation including *Cd163*, *Ccl13*, *Cxcl5*, and *Ccr1.*

From the gene expression data of mouse liver, we identified 45 probes. After eliminating the duplicated probes, probes for 30 genes were used for the analysis of the gene network.

In liver, there are only two groups of genes that showed associations. Among profibrotic/*Tgfβ*-regulated genes, *Col14a1*, *Col3a1*, *Col1a1*, and *Spp1* are positively associated but *Col5a2* and *Tgfβ* are not associated with any gene in the profibrotic/*Tgfβ*-regulated pathway. Among *IFN*-regulated genes, *Mx1*, *Oas2*, and *Ifi44* are associated together as one group.

The comparison again indicates that there are some differences between the molecular pathways of the lung and liver. The association among genes in the lung is stronger than that in the liver.

### Gene association of systemic sclerosis-related genes in the literature for human and mouse

We searched the literature for association in publications of these genes in humans and in mice using GeneNetwork. The function of the literature graph provides correlations between two genes based on the frequency of the two genes in the same publication. Thus, the more two genes appear in the same literature, the stronger the correlation of these two genes appears in the literature graph. From the literature report, we did not find any correlation coefficients >0.35 or <−0.35 among the genes from human lung and liver, suggesting that our analysis is novel (Additional file 4: Figure S2).

**Fig. 3:**
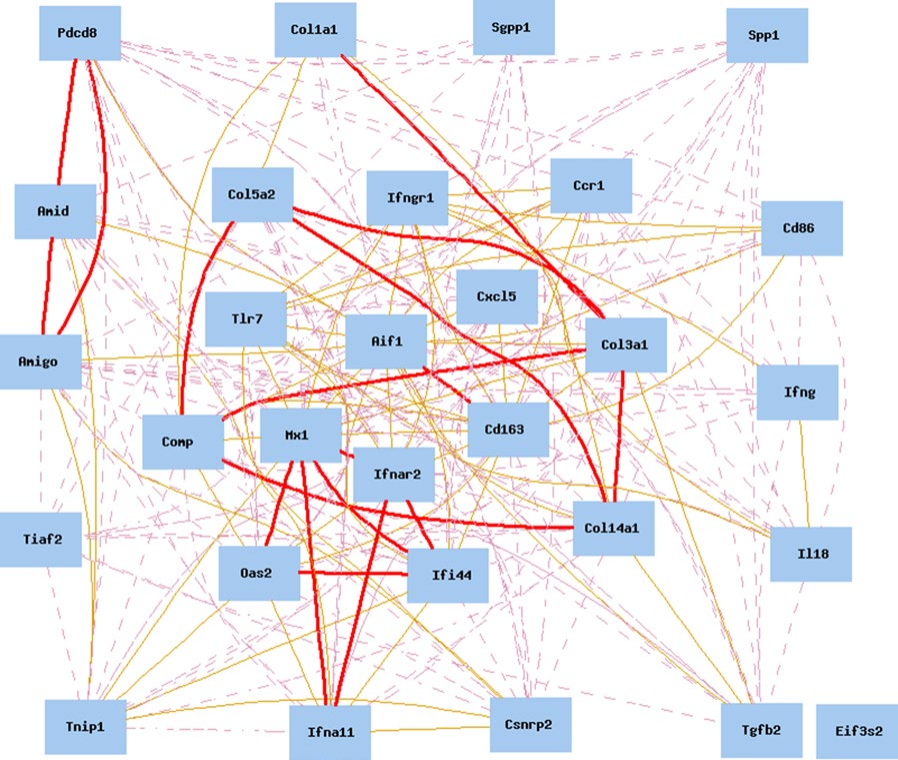
Association of SSc-related genes in literature in mouse model. The 29 nodes in the graph below show the selected traits. All nodes are displayed. The 253 edges between the nodes, filtered from the 406 total edges are drawn as curves, show *Literature* correlation coefficients >0.35 or <−0.35. The graph’s canvas is 40.0 by 40.0 cm, and the node labels are drawn with a 10.0 point font, and the edge labels are drawn with a 10.0 point font

The results from mice, however, indicate that there are strong associations among two groups of genes (Fig. 3). The first group of strongly associated genes includes genes *Col14a1*, *Col3a1*, *Col1a1*, *Col5a2*, and *Comp*, which are known to belong to the Profibrotic/*Tgfβ*-regulated pathway. The second group of strongly associated genes includes *Mx1*, *Oas2*, *Ifi44*, *Ifnar2*, and *Ifna11*, which belong to the *IFN*-regulated pathway. The association of these genes reported in the literature in general agrees with the gene network from the mouse study.

### Gene network of systemic sclerosis-related genes in lung based on data of RNA seq in humans

**Fig. 4:**
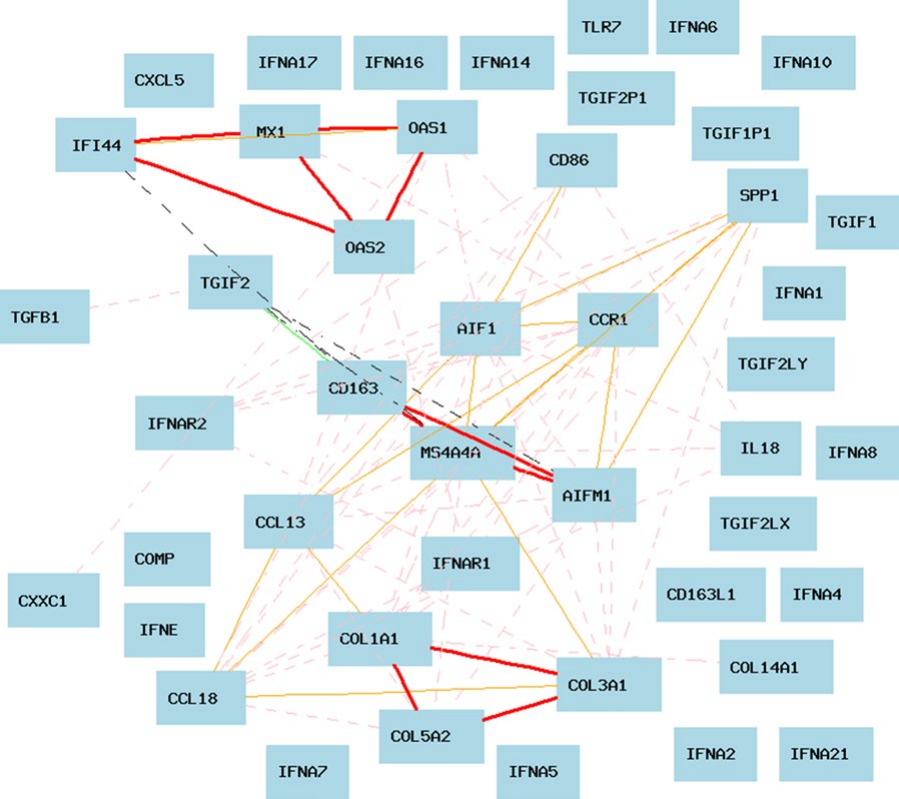
Confirmation of gene network of SSc-related genes in humans with data of RNA seq. Curves show Pearson correlation coefficients >0.35 or <−0.35. *Colors* of *lines* represent different R values. *Red* 0.7–1, *pink* 0.5–0.7, *grey pink* 0–0.5, *black* −0.5 to 0, *green* −0.7 to −0.5, *blue* −1 to −0.7. Gene network of 45 SSc-related genes in lung of humans. The graph’s canvas is 40.0 by 40.0 cm, and the node labels are drawn with a 10.0 point font, and the edge labels are drawn with a 10.0 point font

We searched the GTEx Human Lung (Mar14) RPKM Log2 Database for all records that match the 23 genes using GeneNetwork. We found a total of 45 records which supports the major conclusion of data from the microarray study. Genes for macrophage activation genes (CD163, AIF1, CD86, MS4A4A, CCL18, CCL13, and CCR1) are positively associated, most of them with R values >0.7. CXCL5, however, showed no association with any of these genes (Fig. 4).

Profibrotic/Tgfβ-regulated genes including Col5a2, Col1a1, and Col3a1 are strongly positively associated. COMP showed no association with any of the three groups of genes noted above. However, SPP1 was only weakly positively associated with Col3a1, but had a stronger positive association with AIF1, AIFM1, Col3a1, MS4A4A, CCR1, and CCL13.

Among IFN-regulated genes, MX1, OAS1, IFI44, and OAS2 associated together. There is no direct regulation of this group of genes by either IFN or IFNAR2.

## Discussion

Our analysis revealed potential molecular pathways for SSc-related progressive lung fibrosis. Like other lung fibrosis, SSc-related progressive lung fibrosis arises from a series of molecular and pathological events. Understanding of the molecular basis of SSc-related progressive lung fibrosis is a key in prevention and treatment of the disease. Our data are the first to show the potential molecular pathways of these genes for this disease. The publication of Christmann’s group identified the differentially expressed genes and categorized them [3]. Our study organized them into pathways and connected them together. There are studies on the pathways of these genes in animal models, however, not in humans, mainly due to the limited number of samples. Detailed studies in the future of the order of events, and sequence regulation of events related to expression of these genes will provide information for identification of potential molecular targets in drug design.

There is a difference between humans and mice in the regulation of genes identified in SSc-related progressive lung fibrosis. The macrophage activation genes are associated more strongly in humans than that in mice. Several genes (including CD163, AIF1, CD86, MS4A4A, CCL18, CCL13, and CCR1) are positively associated in humans, while in mice there is a relatively weak association among Cd86 and Aif1. While the association in gene expression between humans and mice are similar for IFN-regulated genes and profibrotic/Tgfβ-regulated genes, the difference between humans and mice on the regulation of macrophage activation genes alerts a potential problematic issue in the translation of research results from the mouse model into the human population. A typical scenario is that treatment in humans targeting mainly macrophage activation genes may be a better antifibrotic strategy; while, in mice, targeting profibrotic/Tgfβ-regulated genes may be a better strategy.

In both humans and mice, the associations among the investigated genes are stronger in the lung than that in the liver. Such a result indicates that, if one of these genes in these gene groups of strong association is dysregulated, it affects the lung more seriously than the liver, because the dysregulated gene may not (in turn) affect the expression levels of others in the liver. The difference of gene associations between lung and liver also supports the reliability of this study. It would be difficult to explain why fibrosis occurs in SSc lungs but not liver, if a stronger association of these genes was found in liver.

In a study using lung tissues from Systemic Sclerosis patients with Pulmonary Fibrosis and Pulmonary Hypertension, Hsu et al. [11] identified 242 and 335 genes that were differentially expressed in lungs and primary fibroblasts, respectively. Our study analyzed the genes from a study of systemic sclerosis-related progressive lung fibrosis and compare the molecular pathways in the lung of normal mouse and human populations. While key genes in both studies, such as COL1A1, TGFb1, are the same, there is a considerable difference in the gene lists between these two studies. Most of the genes in the report by Hsu et al. are not listed in the report by Christmann et al. [3]. Many of the genes in our study involve in the protein bolding, such as COL1A1, COL5A2, COL14A1, CD86, CD163, COMP. They are also the important components for extracellular region. Several genes participate cytokine activity, such as CXCL5, CCL13, CCL18, SPP1, IL18, and IFNA2. It may be necessary for the future to investigate their association with other pathways such as in the insulin-like growth factor signaling and caveolin mediated endocytosis pathways.

The selection of genes in this study is based on data from humans. Several studies suggest that mice have different macrophage markers and it is even organ/model dependent [12–14]. For example, Arginase-1 (Arg1) has been used in animal models as the molecular marker for SSc-Related Progressive Lung Fibrosis [15, 16]. However, Arg1 is not selected as a differentially expressed gene in SSc-Related Progressive Lung Fibrosis in humans. Our data emphasize the importance of identification of similarities and differences between humans and animal models. When applying the results from mouse models to humans, these differences need to be carefully examined to ensure the clinically translational clinic study is oriented in the correct direction.

In studying molecular pathways from mice, we used data from mouse RI strains, which are homozygous in genomics within each of such strains. Evidently the data are accurate not only due to the homozygousity of the mouse strain but also the reproducibility of results from multiple mice within each strain. However, the human population included data from more than 1000 individuals. The gene network, constructed with data from RNAseq, also confirmed the conclusion of the microarray data. Although the results from this study may need further confirmation by additional studies, the data are considerably reliable. We realized that our data were from normal tissues. The observed correlations (or lack of correlation) might change upon TGF-beta or interferon activation in human SSc tissues or murine fibrotic models.

## Additional files

10.1186/s13104-015-1510-4 Genes used for final analysis in human lung.
10.1186/s13104-015-1510-4 Gene network of 82 probes in human lung.
10.1186/s13104-015-1510-4 Genes used for final analysis in human liver.
10.1186/s13104-015-1510-4 No gene network connection in lung human in literature report.
