# Supplementary material for: Molecular network of important genes for systemic sclerosis-related progressive lung fibrosis
Source: BMC Res Notes. 2015 Oct 7;8:544. doi: 10.1186/s13104-015-1510-4 (PMC4596290; doi:10.1186/s13104-015-1510-4)
Supplement: Supplementary file 1 — 10.1186/s13104-015-1510-4 Genes used for final analysis in human lung. [file 13104_2015_1510_MOESM1_ESM.docx]

|  | Dataset 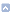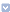 | Trait ID 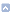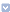 | Symbol 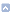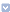 | Description 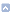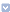 | Location 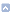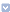 | Mean 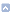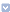 | N Cases 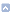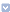 | Max LRS 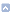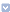 | Max LRS Location Chr and Mb 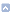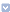 |
| --- | --- | --- | --- | --- | --- | --- | --- | --- | --- |
| 1  | GSE23546HLT0613 | [100140033_TGI_at](javascript:showDatabase3('showDatabase','GSE23546HLT0613','100140033_TGI_at','')) | [AIF1](http://www.ncbi.nlm.nih.gov/entrez/query.fcgi?db=gene&cmd=Retrieve&dopt=Graphics&list_uids=199) | allograft inflammatory factor 1 | Chr6: 31.582994 | 5.173 | 1230 | -- | -- |
| 2  | GSE23546HLT0613 | [100151160_TGI_at](javascript:showDatabase3('showDatabase','GSE23546HLT0613','100151160_TGI_at','')) | [AIF1](http://www.ncbi.nlm.nih.gov/entrez/query.fcgi?db=gene&cmd=Retrieve&dopt=Graphics&list_uids=199) | allograft inflammatory factor 1 | Chr6: 31.582994 | 12.326 | 1230 | -- | -- |
| 3  | GSE23546HLT0613 | [100130966_TGI_at](javascript:showDatabase3('showDatabase','GSE23546HLT0613','100130966_TGI_at','')) | [AIFM1](http://www.ncbi.nlm.nih.gov/entrez/query.fcgi?db=gene&cmd=Retrieve&dopt=Graphics&list_uids=9131) | apoptosis-inducing factor, mitochondrion-associated, 1 | ChrX: 129.263337 | 9.997 | 1230 | -- | -- |
| 4  | GSE23546HLT0613 | [100159583_TGI_at](javascript:showDatabase3('showDatabase','GSE23546HLT0613','100159583_TGI_at','')) | [CCL13](http://www.ncbi.nlm.nih.gov/entrez/query.fcgi?db=gene&cmd=Retrieve&dopt=Graphics&list_uids=6357) | chemokine (C-C motif) ligand 13 | Chr17: 32.683471 | 10.894 | 1230 | -- | -- |
| 5  | GSE23546HLT0613 | [100136911_TGI_at](javascript:showDatabase3('showDatabase','GSE23546HLT0613','100136911_TGI_at','')) | [CCL18](http://www.ncbi.nlm.nih.gov/entrez/query.fcgi?db=gene&cmd=Retrieve&dopt=Graphics&list_uids=6362) | chemokine (C-C motif) ligand 18 (pulmonary and activation-regulated) | Chr17: 34.391643 | 12.281 | 1230 | -- | -- |
| 6  | GSE23546HLT0613 | [100303033_TGI_at](javascript:showDatabase3('showDatabase','GSE23546HLT0613','100303033_TGI_at','')) | [CCR1](http://www.ncbi.nlm.nih.gov/entrez/query.fcgi?db=gene&cmd=Retrieve&dopt=Graphics&list_uids=1230) | chemokine (C-C motif) receptor 1 | Chr3: 46.243200 | 10.741 | 1230 | -- | -- |
| 7  | GSE23546HLT0613 | [100300043_TGI_at](javascript:showDatabase3('showDatabase','GSE23546HLT0613','100300043_TGI_at','')) | [CD163](http://www.ncbi.nlm.nih.gov/entrez/query.fcgi?db=gene&cmd=Retrieve&dopt=Graphics&list_uids=9332) | CD163 molecule | Chr12: 7.623412 | 11.779 | 1230 | -- | -- |
| 8  | GSE23546HLT0613 | [100153378_TGI_at](javascript:showDatabase3('showDatabase','GSE23546HLT0613','100153378_TGI_at','')) | [CD163L1](http://www.ncbi.nlm.nih.gov/entrez/query.fcgi?db=gene&cmd=Retrieve&dopt=Graphics&list_uids=283316) | CD163 molecule-like 1 | Chr12: 7.507556 | 8.231 | 1230 | -- | -- |
| 9  | GSE23546HLT0613 | [100154029_TGI_at](javascript:showDatabase3('showDatabase','GSE23546HLT0613','100154029_TGI_at','')) | [CD86](http://www.ncbi.nlm.nih.gov/entrez/query.fcgi?db=gene&cmd=Retrieve&dopt=Graphics&list_uids=942) | CD86 molecule | Chr3: 121.774209 | 9.815 | 1230 | -- | -- |
| 10  | GSE23546HLT0613 | [100300592_TGI_at](javascript:showDatabase3('showDatabase','GSE23546HLT0613','100300592_TGI_at','')) | [CD86](http://www.ncbi.nlm.nih.gov/entrez/query.fcgi?db=gene&cmd=Retrieve&dopt=Graphics&list_uids=942) | CD86 molecule | Chr3: 121.774209 | 9.420 | 1230 | -- | -- |
| 11  | GSE23546HLT0613 | [100156990_TGI_at](javascript:showDatabase3('showDatabase','GSE23546HLT0613','100156990_TGI_at','')) | [COL14A1](http://www.ncbi.nlm.nih.gov/entrez/query.fcgi?db=gene&cmd=Retrieve&dopt=Graphics&list_uids=7373) | collagen, type XIV, alpha 1 | Chr8: 121.137352 | 10.481 | 1230 | -- | -- |
| 12  | GSE23546HLT0613 | [100147092_TGI_at](javascript:showDatabase3('showDatabase','GSE23546HLT0613','100147092_TGI_at','')) | [COL1A1](http://www.ncbi.nlm.nih.gov/entrez/query.fcgi?db=gene&cmd=Retrieve&dopt=Graphics&list_uids=1277) | collagen, type I, alpha 1 | Chr17: 48.261457 | 12.275 | 1230 | -- | -- |
| 13  | GSE23546HLT0613 | [100159693_TGI_at](javascript:showDatabase3('showDatabase','GSE23546HLT0613','100159693_TGI_at','')) | [COL5A2](http://www.ncbi.nlm.nih.gov/entrez/query.fcgi?db=gene&cmd=Retrieve&dopt=Graphics&list_uids=1290) | collagen, type V, alpha 2 | Chr2: 189.896641 | 9.198 | 1230 | -- | -- |
| 14  | GSE23546HLT0613 | [100149643_TGI_at](javascript:showDatabase3('showDatabase','GSE23546HLT0613','100149643_TGI_at','')) | [COMP](http://www.ncbi.nlm.nih.gov/entrez/query.fcgi?db=gene&cmd=Retrieve&dopt=Graphics&list_uids=1311) | cartilage oligomeric matrix protein | Chr19: 18.893583 | 8.558 | 1230 | -- | -- |
| 15  | GSE23546HLT0613 | [100152679_TGI_at](javascript:showDatabase3('showDatabase','GSE23546HLT0613','100152679_TGI_at','')) | [CXCL5](http://www.ncbi.nlm.nih.gov/entrez/query.fcgi?db=gene&cmd=Retrieve&dopt=Graphics&list_uids=6374) | chemokine (C-X-C motif) ligand 5 | Chr4: 74.861359 | 8.736 | 1230 | -- | -- |
| 16  | GSE23546HLT0613 | [100149436_TGI_at](javascript:showDatabase3('showDatabase','GSE23546HLT0613','100149436_TGI_at','')) | [CXXC1](http://www.ncbi.nlm.nih.gov/entrez/query.fcgi?db=gene&cmd=Retrieve&dopt=Graphics&list_uids=30827) | CXXC finger protein 1 | Chr18: 47.808713 | 9.268 | 1230 | -- | -- |
| 17  | GSE23546HLT0613 | [100137744_TGI_at](javascript:showDatabase3('showDatabase','GSE23546HLT0613','100137744_TGI_at','')) | [IFI44](http://www.ncbi.nlm.nih.gov/entrez/query.fcgi?db=gene&cmd=Retrieve&dopt=Graphics&list_uids=10561) | interferon-induced protein 44 | Chr1: 79.115477 | 10.235 | 1230 | -- | -- |
| 18  | GSE23546HLT0613 | [100126008_TGI_at](javascript:showDatabase3('showDatabase','GSE23546HLT0613','100126008_TGI_at','')) | [IFNA1](http://www.ncbi.nlm.nih.gov/entrez/query.fcgi?db=gene&cmd=Retrieve&dopt=Graphics&list_uids=3439) | interferon, alpha 1 | Chr9: 21.440453 | 4.801 | 1230 | -- | -- |
| 19  | GSE23546HLT0613 | [100129327_TGI_at](javascript:showDatabase3('showDatabase','GSE23546HLT0613','100129327_TGI_at','')) | [IFNA10](http://www.ncbi.nlm.nih.gov/entrez/query.fcgi?db=gene&cmd=Retrieve&dopt=Graphics&list_uids=3446) | interferon, alpha 10 | Chr9: 21.206180 | 5.147 | 1230 | -- | -- |
| 20  | GSE23546HLT0613 | [100154876_TGI_at](javascript:showDatabase3('showDatabase','GSE23546HLT0613','100154876_TGI_at','')) | [IFNA14](http://www.ncbi.nlm.nih.gov/entrez/query.fcgi?db=gene&cmd=Retrieve&dopt=Graphics&list_uids=3448) | interferon, alpha 14 | Chr9: 21.239201 | 4.858 | 1230 | -- | -- |
| 21  | GSE23546HLT0613 | [100124500_TGI_at](javascript:showDatabase3('showDatabase','GSE23546HLT0613','100124500_TGI_at','')) | [IFNA16](http://www.ncbi.nlm.nih.gov/entrez/query.fcgi?db=gene&cmd=Retrieve&dopt=Graphics&list_uids=3449) | interferon, alpha 16 | Chr9: 21.216372 | 4.799 | 1230 | -- | -- |
| 22  | GSE23546HLT0613 | [100132651_TGI_at](javascript:showDatabase3('showDatabase','GSE23546HLT0613','100132651_TGI_at','')) | [IFNA2](http://www.ncbi.nlm.nih.gov/entrez/query.fcgi?db=gene&cmd=Retrieve&dopt=Graphics&list_uids=3440) | interferon, alpha 2 | Chr9: 21.384254 | 5.031 | 1230 | -- | -- |
| 23  | GSE23546HLT0613 | [100147912_TGI_at](javascript:showDatabase3('showDatabase','GSE23546HLT0613','100147912_TGI_at','')) | [IFNA21](http://www.ncbi.nlm.nih.gov/entrez/query.fcgi?db=gene&cmd=Retrieve&dopt=Graphics&list_uids=3452) | interferon, alpha 21 | Chr9: 21.165636 | 5.082 | 1230 | -- | -- |
| 24  | GSE23546HLT0613 | [100152053_TGI_at](javascript:showDatabase3('showDatabase','GSE23546HLT0613','100152053_TGI_at','')) | [IFNA4](http://www.ncbi.nlm.nih.gov/entrez/query.fcgi?db=gene&cmd=Retrieve&dopt=Graphics&list_uids=3441) | interferon, alpha 4 | Chr9: 21.186617 | 5.206 | 1230 | -- | -- |
| 25  | GSE23546HLT0613 | [100124695_TGI_at](javascript:showDatabase3('showDatabase','GSE23546HLT0613','100124695_TGI_at','')) | [IFNA5](http://www.ncbi.nlm.nih.gov/entrez/query.fcgi?db=gene&cmd=Retrieve&dopt=Graphics&list_uids=3442) | interferon, alpha 5 | Chr9: 21.304613 | 4.998 | 1230 | -- | -- |
| 26  | GSE23546HLT0613 | [100159500_TGI_at](javascript:showDatabase3('showDatabase','GSE23546HLT0613','100159500_TGI_at','')) | [IFNA6](http://www.ncbi.nlm.nih.gov/entrez/query.fcgi?db=gene&cmd=Retrieve&dopt=Graphics&list_uids=3443) | interferon, alpha 6 | Chr9: 21.350317 | 5.783 | 1230 | -- | -- |
| 27  | GSE23546HLT0613 | [100155333_TGI_at](javascript:showDatabase3('showDatabase','GSE23546HLT0613','100155333_TGI_at','')) | [IFNA7](http://www.ncbi.nlm.nih.gov/entrez/query.fcgi?db=gene&cmd=Retrieve&dopt=Graphics&list_uids=3444) | interferon, alpha 7 | Chr9: 21.201468 | 4.592 | 1230 | -- | -- |
| 28  | GSE23546HLT0613 | [100144400_TGI_at](javascript:showDatabase3('showDatabase','GSE23546HLT0613','100144400_TGI_at','')) | [IFNA8](http://www.ncbi.nlm.nih.gov/entrez/query.fcgi?db=gene&cmd=Retrieve&dopt=Graphics&list_uids=3445) | interferon, alpha 8 | Chr9: 21.409146 | 5.850 | 1230 | -- | -- |
| 29  | GSE23546HLT0613 | [100305759_TGI_at](javascript:showDatabase3('showDatabase','GSE23546HLT0613','100305759_TGI_at','')) | [IFNAR1](http://www.ncbi.nlm.nih.gov/entrez/query.fcgi?db=gene&cmd=Retrieve&dopt=Graphics&list_uids=3454) | interferon (alpha, beta and omega) receptor 1 | Chr21: 34.697214 | 10.544 | 1230 | -- | -- |
| 30  | GSE23546HLT0613 | [100156729_TGI_at](javascript:showDatabase3('showDatabase','GSE23546HLT0613','100156729_TGI_at','')) | [IFNAR2](http://www.ncbi.nlm.nih.gov/entrez/query.fcgi?db=gene&cmd=Retrieve&dopt=Graphics&list_uids=3455) | interferon (alpha, beta and omega) receptor 2 | Chr21: 34.602231 | 10.629 | 1230 | -- | -- |
| 31  | GSE23546HLT0613 | [100162928_TGI_at](javascript:showDatabase3('showDatabase','GSE23546HLT0613','100162928_TGI_at','')) | [IFNE](http://www.ncbi.nlm.nih.gov/entrez/query.fcgi?db=gene&cmd=Retrieve&dopt=Graphics&list_uids=338376) | interferon, epsilon | Chr9: 21.480838 | 5.474 | 1230 | -- | -- |
| 32  | GSE23546HLT0613 | [100139153_TGI_at](javascript:showDatabase3('showDatabase','GSE23546HLT0613','100139153_TGI_at','')) | [IL18](http://www.ncbi.nlm.nih.gov/entrez/query.fcgi?db=gene&cmd=Retrieve&dopt=Graphics&list_uids=3606) | interleukin 18 (interferon-gamma-inducing factor) | Chr11: 112.013974 | 9.472 | 1230 | -- | -- |
| 33  | GSE23546HLT0613 | [100159127_TGI_at](javascript:showDatabase3('showDatabase','GSE23546HLT0613','100159127_TGI_at','')) | [MS4A4A](http://www.ncbi.nlm.nih.gov/entrez/query.fcgi?db=gene&cmd=Retrieve&dopt=Graphics&list_uids=51338) | membrane-spanning 4-domains, subfamily A, member 4A | Chr11: 60.048014 | 10.682 | 1230 | -- | -- |
| 34  | GSE23546HLT0613 | [100149348_TGI_at](javascript:showDatabase3('showDatabase','GSE23546HLT0613','100149348_TGI_at','')) | [MX1](http://www.ncbi.nlm.nih.gov/entrez/query.fcgi?db=gene&cmd=Retrieve&dopt=Graphics&list_uids=4599) | myxovirus (influenza virus) resistance 1, interferon-inducible protein p78 (mouse) | Chr21: 42.792520 | 11.486 | 1230 | -- | -- |
| 35  | GSE23546HLT0613 | [100302111_TGI_at](javascript:showDatabase3('showDatabase','GSE23546HLT0613','100302111_TGI_at','')) | [OAS1](http://www.ncbi.nlm.nih.gov/entrez/query.fcgi?db=gene&cmd=Retrieve&dopt=Graphics&list_uids=4938) | 2'-5'-oligoadenylate synthetase 1, 40/46kDa | Chr12: 113.344739 | 9.578 | 1230 | -- | -- |
| 36  | GSE23546HLT0613 | [100302110_TGI_at](javascript:showDatabase3('showDatabase','GSE23546HLT0613','100302110_TGI_at','')) | [OAS2](http://www.ncbi.nlm.nih.gov/entrez/query.fcgi?db=gene&cmd=Retrieve&dopt=Graphics&list_uids=4939) | 2'-5'-oligoadenylate synthetase 2, 69/71kDa | Chr12: 113.416274 | 10.223 | 1230 | -- | -- |
| 37  | GSE23546HLT0613 | [100304472_TGI_at](javascript:showDatabase3('showDatabase','GSE23546HLT0613','100304472_TGI_at','')) | [SPP1](http://www.ncbi.nlm.nih.gov/entrez/query.fcgi?db=gene&cmd=Retrieve&dopt=Graphics&list_uids=6696) | secreted phosphoprotein 1 | Chr4: 88.896802 | 8.806 | 1230 | -- | -- |
| 38  | GSE23546HLT0613 | [100154152_TGI_at](javascript:showDatabase3('showDatabase','GSE23546HLT0613','100154152_TGI_at','')) | [TGFB1](http://www.ncbi.nlm.nih.gov/entrez/query.fcgi?db=gene&cmd=Retrieve&dopt=Graphics&list_uids=7040) | transforming growth factor, beta 1 | Chr19: 41.836812 | 9.624 | 1230 | -- | -- |
| 39  | GSE23546HLT0613 | [100134839_TGI_at](javascript:showDatabase3('showDatabase','GSE23546HLT0613','100134839_TGI_at','')) | [TGIF1](http://www.ncbi.nlm.nih.gov/entrez/query.fcgi?db=gene&cmd=Retrieve&dopt=Graphics&list_uids=7050) | TGFB-induced factor homeobox 1 | Chr18: 3.412072 | 10.986 | 1230 | -- | -- |
| 40  | GSE23546HLT0613 | [100133036_TGI_at](javascript:showDatabase3('showDatabase','GSE23546HLT0613','100133036_TGI_at','')) | [TGIF2](http://www.ncbi.nlm.nih.gov/entrez/query.fcgi?db=gene&cmd=Retrieve&dopt=Graphics&list_uids=60436) | TGFB-induced factor homeobox 2 | Chr20: 35.201876 | 9.546 | 1230 | -- | -- |
| 41  | GSE23546HLT0613 | [100151478_TGI_at](javascript:showDatabase3('showDatabase','GSE23546HLT0613','100151478_TGI_at','')) | [TLR7](http://www.ncbi.nlm.nih.gov/entrez/query.fcgi?db=gene&cmd=Retrieve&dopt=Graphics&list_uids=51284) | toll-like receptor 7 | ChrX: 12.885202 | 9.562 | 1230 | -- | -- |
