# Supplementary figures and images for: Molecular network of important genes for systemic sclerosis-related progressive lung fibrosis
Source: BMC Res Notes. 2015 Oct 7;8:544. doi: 10.1186/s13104-015-1510-4 (PMC4596290; doi:10.1186/s13104-015-1510-4)

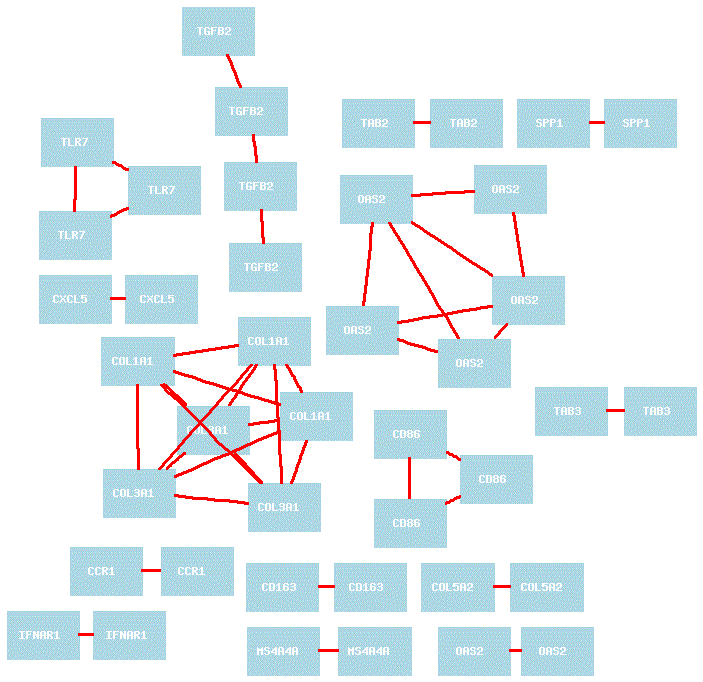

Supplement: Supplementary file 2 — 10.1186/s13104-015-1510-4 Gene network of 82 probes in human lung. [file 13104_2015_1510_MOESM2_ESM.gif]

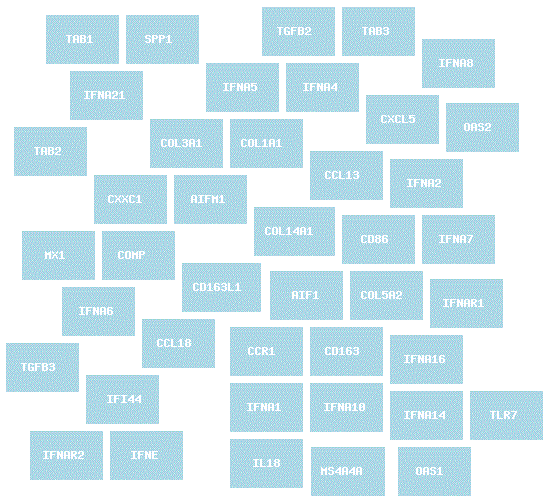

Supplement: Supplementary file 4 — 10.1186/s13104-015-1510-4 No gene network connection in lung human in literature report. [file 13104_2015_1510_MOESM4_ESM.gif]
