# Supplementary material for: Molecular network of important genes for systemic sclerosis-related progressive lung fibrosis
Source: BMC Res Notes. 2015 Oct 7;8:544. doi: 10.1186/s13104-015-1510-4 (PMC4596290; doi:10.1186/s13104-015-1510-4)
Supplement: Supplementary file 3 — 10.1186/s13104-015-1510-4 Genes used for final analysis in human liver. [file 13104_2015_1510_MOESM3_ESM.docx]

|  | Dataset 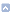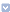 | Trait ID 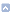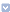 | Symbol 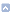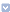 | Description 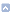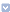 | Location 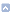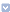 | Mean 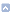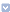 | N Cases 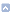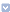 | Max LRS 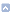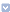 | Max LRS Location Chr and Mb 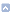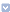 |
| --- | --- | --- | --- | --- | --- | --- | --- | --- | --- |
| 1  | HLC_0311 | [10026391229](javascript:showDatabase3('showDatabase','HLC_0311','10026391229','')) | [AIF1](http://www.ncbi.nlm.nih.gov/entrez/query.fcgi?db=gene&cmd=Retrieve&dopt=Graphics&list_uids=199) | allograft inflammatory factor 1 | Chr6: 3.094416 | -0.034 | 427 | -- | -- |
| 2  | HLC_0311 | [10023804829](javascript:showDatabase3('showDatabase','HLC_0311','10023804829','')) | [AIFM1](http://www.ncbi.nlm.nih.gov/entrez/query.fcgi?db=gene&cmd=Retrieve&dopt=Graphics&list_uids=9131) | apoptosis-inducing factor, mitochondrion-associated, 1 | ChrX: 129.263337 | -0.038 | 427 | -- | -- |
| 3  | HLC_0311 | [10023813366](javascript:showDatabase3('showDatabase','HLC_0311','10023813366','')) | [CCL13](http://www.ncbi.nlm.nih.gov/entrez/query.fcgi?db=gene&cmd=Retrieve&dopt=Graphics&list_uids=6357) | chemokine (C-C motif) ligand 13 | Chr17: 32.685629 | -0.143 | 427 | -- | -- |
| 4  | HLC_0311 | [10025908890](javascript:showDatabase3('showDatabase','HLC_0311','10025908890','')) | [CCL18](http://www.ncbi.nlm.nih.gov/entrez/query.fcgi?db=gene&cmd=Retrieve&dopt=Graphics&list_uids=6362) | chemokine (C-C motif) ligand 18 (pulmonary and activation-regulated) | Chr17: 34.398841 | -0.128 | 427 | -- | -- |
| 5  | HLC_0311 | [10025911800](javascript:showDatabase3('showDatabase','HLC_0311','10025911800','')) | [CCR1](http://www.ncbi.nlm.nih.gov/entrez/query.fcgi?db=gene&cmd=Retrieve&dopt=Graphics&list_uids=1230) | chemokine (C-C motif) receptor 1 | Chr3: 46.243199 | -0.113 | 427 | -- | -- |
| 6  | HLC_0311 | [10025904637](javascript:showDatabase3('showDatabase','HLC_0311','10025904637','')) | [CD163](http://www.ncbi.nlm.nih.gov/entrez/query.fcgi?db=gene&cmd=Retrieve&dopt=Graphics&list_uids=9332) | CD163 molecule | Chr12: 7.623409 | -0.118 | 427 | -- | -- |
| 7  | HLC_0311 | [10025903112](javascript:showDatabase3('showDatabase','HLC_0311','10025903112','')) | [CD163L1](http://www.ncbi.nlm.nih.gov/entrez/query.fcgi?db=gene&cmd=Retrieve&dopt=Graphics&list_uids=283316) | CD163 molecule-like 1 | Chr12: 7.507556 | 0.006 | 427 | -- | -- |
| 8  | HLC_0311 | [10023810100](javascript:showDatabase3('showDatabase','HLC_0311','10023810100','')) | [CD86](http://www.ncbi.nlm.nih.gov/entrez/query.fcgi?db=gene&cmd=Retrieve&dopt=Graphics&list_uids=942) | CD86 molecule | Chr3: 121.839983 | -0.046 | 427 | -- | -- |
| 9  | HLC_0311 | [10023815167](javascript:showDatabase3('showDatabase','HLC_0311','10023815167','')) | [COL14A1](http://www.ncbi.nlm.nih.gov/entrez/query.fcgi?db=gene&cmd=Retrieve&dopt=Graphics&list_uids=7373) | collagen, type XIV, alpha 1 (undulin) | Chr8: 121.384273 | -0.021 | 427 | -- | -- |
| 10  | HLC_0311 | [10026392348](javascript:showDatabase3('showDatabase','HLC_0311','10026392348','')) | [COL1A1](http://www.ncbi.nlm.nih.gov/entrez/query.fcgi?db=gene&cmd=Retrieve&dopt=Graphics&list_uids=1277) | collagen, type I, alpha 1 | Chr17: 48.261456 | -0.105 | 427 | -- | -- |
| 11  | HLC_0311 | [10023808730](javascript:showDatabase3('showDatabase','HLC_0311','10023808730','')) | [COL3A1](http://www.ncbi.nlm.nih.gov/entrez/query.fcgi?db=gene&cmd=Retrieve&dopt=Graphics&list_uids=1281) | collagen, type III, alpha 1 (Ehlers-Danlos syndrome type IV, autosomal dominant) | Chr2: 189.877472 | -0.044 | 427 | -- | -- |
| 12  | HLC_0311 | [10023806074](javascript:showDatabase3('showDatabase','HLC_0311','10023806074','')) | [COL5A2](http://www.ncbi.nlm.nih.gov/entrez/query.fcgi?db=gene&cmd=Retrieve&dopt=Graphics&list_uids=1290) | collagen, type V, alpha 2 | Chr2: 189.896640 | 0.044 | 427 | -- | -- |
| 13  | HLC_0311 | [10023812836](javascript:showDatabase3('showDatabase','HLC_0311','10023812836','')) | [COMP](http://www.ncbi.nlm.nih.gov/entrez/query.fcgi?db=gene&cmd=Retrieve&dopt=Graphics&list_uids=1311) | cartilage oligomeric matrix protein | Chr19: 18.893582 | 0.003 | 427 | -- | -- |
| 14  | HLC_0311 | [10025909609](javascript:showDatabase3('showDatabase','HLC_0311','10025909609','')) | [CXCL5](http://www.ncbi.nlm.nih.gov/entrez/query.fcgi?db=gene&cmd=Retrieve&dopt=Graphics&list_uids=6374) | chemokine (C-X-C motif) ligand 5 | Chr4: 74.861358 | -0.073 | 414 | -- | -- |
| 15  | HLC_0311 | [10023820788](javascript:showDatabase3('showDatabase','HLC_0311','10023820788','')) | [CXXC1](http://www.ncbi.nlm.nih.gov/entrez/query.fcgi?db=gene&cmd=Retrieve&dopt=Graphics&list_uids=30827) | CXXC finger 1 (PHD domain) | Chr18: 47.808712 | 0.008 | 427 | -- | -- |
| 16  | HLC_0311 | [10023810338](javascript:showDatabase3('showDatabase','HLC_0311','10023810338','')) | [IFI44](http://www.ncbi.nlm.nih.gov/entrez/query.fcgi?db=gene&cmd=Retrieve&dopt=Graphics&list_uids=10561) | interferon-induced protein 44 | Chr1: 79.129763 | -0.070 | 427 | -- | -- |
| 17  | HLC_0311 | [10025911167](javascript:showDatabase3('showDatabase','HLC_0311','10025911167','')) | [IFNA1](http://www.ncbi.nlm.nih.gov/entrez/query.fcgi?db=gene&cmd=Retrieve&dopt=Graphics&list_uids=3439) | interferon, alpha 1 | Chr9: 21.441315 | -0.034 | 422 | -- | -- |
| 18  | HLC_0311 | [10025905014](javascript:showDatabase3('showDatabase','HLC_0311','10025905014','')) | [IFNA10](http://www.ncbi.nlm.nih.gov/entrez/query.fcgi?db=gene&cmd=Retrieve&dopt=Graphics&list_uids=3446) | interferon, alpha 10 | Chr9: 21.206179 | -0.069 | 412 | -- | -- |
| 19  | HLC_0311 | [10025908713](javascript:showDatabase3('showDatabase','HLC_0311','10025908713','')) | [IFNA17](http://www.ncbi.nlm.nih.gov/entrez/query.fcgi?db=gene&cmd=Retrieve&dopt=Graphics&list_uids=3451) | interferon, alpha 17 | Chr9: 21.227241 | 0.000 | 426 | -- | -- |
| 20  | HLC_0311 | [10025908809](javascript:showDatabase3('showDatabase','HLC_0311','10025908809','')) | [IFNA2](http://www.ncbi.nlm.nih.gov/entrez/query.fcgi?db=gene&cmd=Retrieve&dopt=Graphics&list_uids=3440) | interferon, alpha 2 | Chr9: 21.384253 | 0.117 | 425 | -- | -- |
| 21  | HLC_0311 | [10025911845](javascript:showDatabase3('showDatabase','HLC_0311','10025911845','')) | [IFNA21](http://www.ncbi.nlm.nih.gov/entrez/query.fcgi?db=gene&cmd=Retrieve&dopt=Graphics&list_uids=3452) | interferon, alpha 21 | Chr9: 21.165635 | -0.034 | 355 | -- | -- |
| 22  | HLC_0311 | [10025904110](javascript:showDatabase3('showDatabase','HLC_0311','10025904110','')) | [IFNA4](http://www.ncbi.nlm.nih.gov/entrez/query.fcgi?db=gene&cmd=Retrieve&dopt=Graphics&list_uids=3441) | interferon, alpha 4 | Chr9: 21.186617 | -0.052 | 417 | -- | -- |
| 23  | HLC_0311 | [10025904863](javascript:showDatabase3('showDatabase','HLC_0311','10025904863','')) | [IFNA5](http://www.ncbi.nlm.nih.gov/entrez/query.fcgi?db=gene&cmd=Retrieve&dopt=Graphics&list_uids=3442) | interferon, alpha 5 | Chr9: 21.304612 | 0.029 | 427 | -- | -- |
| 24  | HLC_0311 | [10025911429](javascript:showDatabase3('showDatabase','HLC_0311','10025911429','')) | [IFNA6](http://www.ncbi.nlm.nih.gov/entrez/query.fcgi?db=gene&cmd=Retrieve&dopt=Graphics&list_uids=3443) | interferon, alpha 6 | Chr9: 21.350316 | 0.303 | 426 | -- | -- |
| 25  | HLC_0311 | [10025903382](javascript:showDatabase3('showDatabase','HLC_0311','10025903382','')) | [IFNA7](http://www.ncbi.nlm.nih.gov/entrez/query.fcgi?db=gene&cmd=Retrieve&dopt=Graphics&list_uids=3444) | interferon, alpha 7 | Chr9: 21.201467 | -0.038 | 427 | -- | -- |
| 26  | HLC_0311 | [10025912215](javascript:showDatabase3('showDatabase','HLC_0311','10025912215','')) | [IFNA8](http://www.ncbi.nlm.nih.gov/entrez/query.fcgi?db=gene&cmd=Retrieve&dopt=Graphics&list_uids=3445) | interferon, alpha 8 | Chr9: 21.410184 | -0.111 | 427 | -- | -- |
| 27  | HLC_0311 | [10025909987](javascript:showDatabase3('showDatabase','HLC_0311','10025909987','')) | [IFNAR1](http://www.ncbi.nlm.nih.gov/entrez/query.fcgi?db=gene&cmd=Retrieve&dopt=Graphics&list_uids=3454) | interferon (alpha, beta and omega) receptor 1 | Chr21: 34.732128 | -0.003 | 427 | -- | -- |
| 28  | HLC_0311 | [10025905549](javascript:showDatabase3('showDatabase','HLC_0311','10025905549','')) | [IFNAR2](http://www.ncbi.nlm.nih.gov/entrez/query.fcgi?db=gene&cmd=Retrieve&dopt=Graphics&list_uids=3455) | interferon (alpha, beta and omega) receptor 2 | Chr21: 34.635062 | -0.033 | 427 | -- | -- |
| 29  | HLC_0311 | [10025909785](javascript:showDatabase3('showDatabase','HLC_0311','10025909785','')) | [IFNE1](http://www.ncbi.nlm.nih.gov/entrez/query.fcgi?db=gene&cmd=Retrieve&dopt=Graphics&list_uids=338376) | interferon epsilon 1 | Chr9: 21.480838 | -0.012 | 408 | -- | -- |
| 30  | HLC_0311 | [10023804764](javascript:showDatabase3('showDatabase','HLC_0311','10023804764','')) | [IL18](http://www.ncbi.nlm.nih.gov/entrez/query.fcgi?db=gene&cmd=Retrieve&dopt=Graphics&list_uids=3606) | interleukin 18 (interferon-gamma-inducing factor) | Chr11: 112.013975 | -0.062 | 427 | -- | -- |
| 31  | HLC_0311 | [10023804739](javascript:showDatabase3('showDatabase','HLC_0311','10023804739','')) | [MS4A4A](http://www.ncbi.nlm.nih.gov/entrez/query.fcgi?db=gene&cmd=Retrieve&dopt=Graphics&list_uids=51338) | membrane-spanning 4-domains, subfamily A, member 4 | Chr11: 60.076445 | -0.100 | 427 | -- | -- |
| 32  | HLC_0311 | [10025909781](javascript:showDatabase3('showDatabase','HLC_0311','10025909781','')) | [MS4A4A](http://www.ncbi.nlm.nih.gov/entrez/query.fcgi?db=gene&cmd=Retrieve&dopt=Graphics&list_uids=51338) | membrane-spanning 4-domains, subfamily A, member 4 | Chr11: 60.076445 | -0.016 | 427 | -- | -- |
| 33  | HLC_0311 | [10025902733](javascript:showDatabase3('showDatabase','HLC_0311','10025902733','')) | [MX1](http://www.ncbi.nlm.nih.gov/entrez/query.fcgi?db=gene&cmd=Retrieve&dopt=Graphics&list_uids=4599) | myxovirus (influenza virus) resistance 1, interferon-inducible protein p78 (mouse) | Chr21: 42.831141 | -0.116 | 427 | -- | -- |
| 34  | HLC_0311 | [10025910988](javascript:showDatabase3('showDatabase','HLC_0311','10025910988','')) | [OAS1](http://www.ncbi.nlm.nih.gov/entrez/query.fcgi?db=gene&cmd=Retrieve&dopt=Graphics&list_uids=4938) | 2',5'-oligoadenylate synthetase 1, 40/46kDa | Chr12: 113.355831 | -0.162 | 427 | -- | -- |
| 35  | HLC_0311 | [10025906224](javascript:showDatabase3('showDatabase','HLC_0311','10025906224','')) | [OAS2](http://www.ncbi.nlm.nih.gov/entrez/query.fcgi?db=gene&cmd=Retrieve&dopt=Graphics&list_uids=4939) | 2'-5'-oligoadenylate synthetase 2, 69/71kDa | Chr12: 113.448503 | 0.015 | 427 | -- | -- |
| 36  | HLC_0311 | [10023806479](javascript:showDatabase3('showDatabase','HLC_0311','10023806479','')) | [SPP1](http://www.ncbi.nlm.nih.gov/entrez/query.fcgi?db=gene&cmd=Retrieve&dopt=Graphics&list_uids=6696) | secreted phosphoprotein 1 (osteopontin, bone sialoprotein I, early T-lymphocyte activation 1) | Chr4: 88.904563 | -0.046 | 427 | -- | -- |
| 37  | HLC_0311 | [10025913303](javascript:showDatabase3('showDatabase','HLC_0311','10025913303','')) | [TGFB1](http://www.ncbi.nlm.nih.gov/entrez/query.fcgi?db=gene&cmd=Retrieve&dopt=Graphics&list_uids=7040) | transforming growth factor, beta 1 | Chr19: 41.836811 | 0.048 | 427 | -- | -- |
| 38  | HLC_0311 | [10025905169](javascript:showDatabase3('showDatabase','HLC_0311','10025905169','')) | [TGIF1](http://www.ncbi.nlm.nih.gov/entrez/query.fcgi?db=gene&cmd=Retrieve&dopt=Graphics&list_uids=7050) | TGFB-induced factor homeobox 1 | Chr18: 3.458406 | 0.012 | 427 | -- | -- |
| 39  | HLC_0311 | [10025902622](javascript:showDatabase3('showDatabase','HLC_0311','10025902622','')) | [TGIF2](http://www.ncbi.nlm.nih.gov/entrez/query.fcgi?db=gene&cmd=Retrieve&dopt=Graphics&list_uids=60436) | TGFB-induced factor homeobox 2 | Chr20: 35.222355 | -0.005 | 427 | -- | -- |
| 40  | HLC_0311 | [10025912769](javascript:showDatabase3('showDatabase','HLC_0311','10025912769','')) | [TGIF2LX](http://www.ncbi.nlm.nih.gov/entrez/query.fcgi?db=gene&cmd=Retrieve&dopt=Graphics&list_uids=90316) | TGFB-induced factor homeobox 2-like, X-linked | ChrX: 89.177882 | 0.038 | 427 | -- | -- |
| 41  | HLC_0311 | [10025906618](javascript:showDatabase3('showDatabase','HLC_0311','10025906618','')) | [TGIF2LY](http://www.ncbi.nlm.nih.gov/entrez/query.fcgi?db=gene&cmd=Retrieve&dopt=Graphics&list_uids=90655) | TGFB-induced factor homeobox 2-like, Y-linked | ChrY: 3.448082 | 0.015 | 427 | -- | -- |
| 42  | HLC_0311 | [10025908882](javascript:showDatabase3('showDatabase','HLC_0311','10025908882','')) | [TLR7](http://www.ncbi.nlm.nih.gov/entrez/query.fcgi?db=gene&cmd=Retrieve&dopt=Graphics&list_uids=51284) | toll-like receptor 7 | ChrX: 12.908480 | 0.025 | 427 | -- | -- |
